# Analysis of the Trends of Methicillin-Resistant Staphylococcus aureus in Gauteng Public Hospitals from 2009 to 2018

**DOI:** 10.1128/spectrum.03623-22

**Published:** 2023-06-20

**Authors:** Bradley Segal, Alice Langham, Rachel Klevansky, Namita Patel, Thabang Mokoena, Muhammad Nassiep, Obakeng Ramatlo, Aijaz Ahmad, Adriano G. Duse

**Affiliations:** a Department of Clinical Microbiology and Infectious Diseases, School of Pathology, Faculty of Health Sciences, University of the Witwatersrand, Johannesburg, South Africa; b Department of Clinical Medicine, Faculty of Health Sciences, University of the Witwatersrand, Johannesburg, South Africa; c Division of Infection Control, Charlotte Maxeke Johannesburg Academic Hospital, National Health Laboratory Service, Johannesburg, South Africa; Riverside University Health System Medical Center University of California

**Keywords:** MRSA, MSSA, prevalence, community-acquired infection, hospital-acquired infection

## Abstract

Most investigations into the distribution of methicillin resistant Staphylococcus aureus (MRSA) have focused exclusively on bloodborne infections within individual health care institutions for shorter time periods. This has limited the analysis of a community-spread pathogen to snapshots within the hospital domain. Therefore, in this study we determined the demographic and geographical patterns of MRSA infections and their fluctuation in 10 years within all public hospitals in Gauteng, South Africa. A retrospective analysis of S. aureus samples was done by deduplicating samples in two groups. The sample groups were placed into subsets with respect to demographic and geographical fields and compared across the studied period. Logistic regression was utilized to determine odds ratios for resistant infections in univariate and multivariable configurations. A total of 66,071 unique infectious events were identified from the 148,065 samples received over a 10-year period, out of which 14,356 were identified as bacteremia. MRSA bacteremia rates in Gauteng peaked in 2015 and have since decreased. Within Gauteng, metropolitan areas have the greatest burden of MRSA with children under 5 years of age and males being most affected. Medical wards have the highest S. aureus bacteremia rates, while intensive care units have the highest MRSA bacteremia rates. Patient age, admitting ward, and geographical district are the most important associated factors of resistance. MRSA acquisition rates have shown tremendous growth since 2009 but have since spiked and subsequently decreased. This may be due to the initiation of the National Guidelines on Antimicrobial Stewardship and Infectious Disease Surveillance. Further studies to determine the trajectory of infections are required to support these claims.

**IMPORTANCE** S. aureus is the leading cause of a variety of devastating clinical conditions, including infective endocarditis, bacteremia, and pleuropulmonary infections. It is an important pathogen responsible for substantial morbidity and mortality. MRSA is a variant of interest originally responsible for difficult to treat hospital-acquired infections that has since achieved community spread throughout the world. Most investigations into the distribution of MRSA have focused exclusively on bloodborne infections within individual health care institutions for shorter periods. This has limited the analysis of a community-spread pathogen to snapshots within the hospital domain. This study sought to determine the demographic and geographical patterns of MRSA infections as well as how these have fluctuated over time within all public hospitals. This will also help in understanding the epidemiology and resistance trends of S. aureus, which will help clinicians to understand the clinical prospective and policy makers to design guidelines and strategies for treating such infections.

## INTRODUCTION

Staphylococcus aureus is both a commensal and a human pathogen and is known to colonize approximately 30% of the human population (1). S. aureus has been reported to cause illnesses ranging from mild skin and soft tissue infections to invasive, life-threatening diseases (2). Being a highly adaptive organism and capable of acquiring resistance to commonly used antibiotics, S. aureus infections cause serious illness, more extended hospital stays, higher risk of death, and significantly higher treatment costs (3, 4). A commonly studied resistance acquired by S. aureus is its methicillin resistance. Methicillin resistance in S. aureus is primarily due to the expression of penicillin-binding protein 2_a_ (PBP2_a_/PBP2′), which is encoded by the *mec*(A) gene of the mobile staphylococcal chromosomal cassette (SCC*mec*) (5). Methicillin-resistant Staphylococcus aureus (MRSA) first emerged in the 1960s, soon after methicillin was introduced into clinical therapeutics. By the end of that decade, it was responsible for hospital outbreaks in Western Europe, Australia, and the United States (6). Globally, the proportion of MRSA to all S. aureus infections increased and peaked at 44.2% in 2008 and has been decreasing since (7). In Africa, most countries are experiencing an increase in the prevalence of MRSA infections, with the only exception being South Africa, which saw a decrease in MRSA prevalence (6). Studies of S. aureus infections in South African hospitals have found around 30% MRSA among all S. aureus infections (8, 9). Between 2007 and 2011, the prevalence of MRSA in South Africa decreased from 36% to 24% (6). This same downward trend was also shown in another study, in which there was a decrease in the number of MRSA isolates in South African hospitals from 2010 to 2012 (10).

A pertinent factor to consider in South Africa is the relationship between HIV and MRSA. People with HIV are 6 to 18 times more likely to contract MRSA infections than the general population, and 6.9% of patients who have HIV are carriers of MRSA (11, 12). Another essential factor to note in South Africa is that MRSA is predominantly hospital-acquired MRSA (HA-MRSA; 26.8%), as opposed to community-acquired MRSA (CA-MRSA; 2.3%) (2, 8). Demographic data reveal that in South Africa, S. aureus has the highest infection rate in the extremes of age, with those under 5 years old and the elderly being at the highest risk (1, 10). The majority of CA-MRSA and HA-MRSA cases occur in patients less than 1 year of age, and most of these are in children less than 28 days old (8). With a male:female ratio of 1.5, S. aureus bacteremia incidence is increased in males, with 60.4% of all MRSA-infected patients being males (1, 8).

Geographic distribution data confirmed that Gauteng hospitals have the highest proportion of MRSA compared to other South African hospitals (10). This trend also extended to the pediatric wards, where Gauteng had the highest prevalence of MRSA infections compared to the Western Cape and other provinces (8). With a closer look in Gauteng, Johannesburg hospitals had a higher incidence of MRSA than the Tshwane hospitals (13).

Despite several studies reporting S. aureus susceptibility to methicillin in South Africa, previous research focused on a small number of hospitals (which were usually academic or tertiary centers) (10). This may have led to inaccurate findings, as many patients are treated outside these centers. Additionally, patients in smaller centers may have different characteristics or presentations to those seen in specialist hospitals. Therefore, this study investigated many patients from various centers within public hospitals in Gauteng over 10 years from 2009 to 2018, covering the trends of MRSA infections and how they were distributed across various demographic and geographical groups.

## RESULTS

### Data collection.

A total of 148,065 records were initially received from the National Health Laboratory Service (NHLS). After removing samples occurring outside the study period (*n* = 9,318) and samples with invalid identification numbers (*n* = 47), related samples were collated by deduplication. The 72,692 isolates of the remaining 138,700 samples were collapsed based on the infectious episode criteria to produce a final set of 66,071 infectious events included in the analysis.

### Methicillin resistance.

Of 66,071 S. aureus isolates selected for this study, 21.73% (*n* = 14,356) of events were isolated from blood cultures and constituted bacteremia. Concerning methicillin resistance, 13.79% (*n* = 9,110) of general and 18.91% (*n* = 2,714) of blood culture S. aureus isolates were MRSA. The average numbers of recurrent cases per patient with MRSA and methicillin-susceptible S. aureus (MSSA) infections were 3.6% (95% confidence interval [CI], 3.2 to 4.0%) and 2.3% (95% CI, 2.2 to 2.4%) of cases, respectively. Recurrent bacteremia episodes for MRSA and MSSA were 1.9% (95% CI, 1.4 to 2.4%) and 2.2% (95% CI, 1.9 to 2.5%) of cases in comparison.

### Demographics.

Data analysis in this study revealed that males contract S. aureus infections at a greater rate than females, irrespective of the site of origin. The cohort properties representing age, sex, and ethnicity of the S. aureus infections are presented in Table 1.

**TABLE 1:** Cohort properties based on age, sex, and ethnicity for S. aureus infections^*a*^

Demographic	General isolates	Blood culture isolates
Sample size	66,071	14,356
Age (median [IQR])	30.00 [7.00, 45.00]	11.00 [0.00, 40.00]
Age group		
Newborn	3,979 (6.0%)	2,558 (17.8%)
Infant	5,392 (8.2%)	2,353 (16.4%)
Preschool child	2,477 (3.7%)	603 (4.2%)
Child	3,216 (4.9%)	510 (3.6%)
Adolescent	1,888 (2.9%)	289 (2.0%)
Adult	21,521 (32.6%)	3,104 (21.6%)
Middle aged	9,866 (14.9%)	1,724 (12.0%)
Aged	3,014 (4.6%)	667 (4.6%)
Aged 80	498 (0.8%)	145 (1.0%)
Unknown	14,220 (21.5%)	2,403 (16.7%)
Sex		
Female	29,002 (43.9%)	5,735 (39.9%)
Male	35,545 (53.8%)	8,085 (56.3%)
Unknown	1,524 (2.3%)	536 (3.7%)
Ethnicity		
African	847 (1.3%)	155 (1.1%)
Colored	5*	2*
Indian	5*	2*
Unknown	65,152 (98.6%)	14,192 (98.9%)
White	62 (0.1%)	5 (0.0%)
No. of MSSA (%)	56,961 (86.2)	11,642 (81.1)

a IQR, interquartile range. *, percentages for these numbers were too low to report in this format.

**FIG 1:**
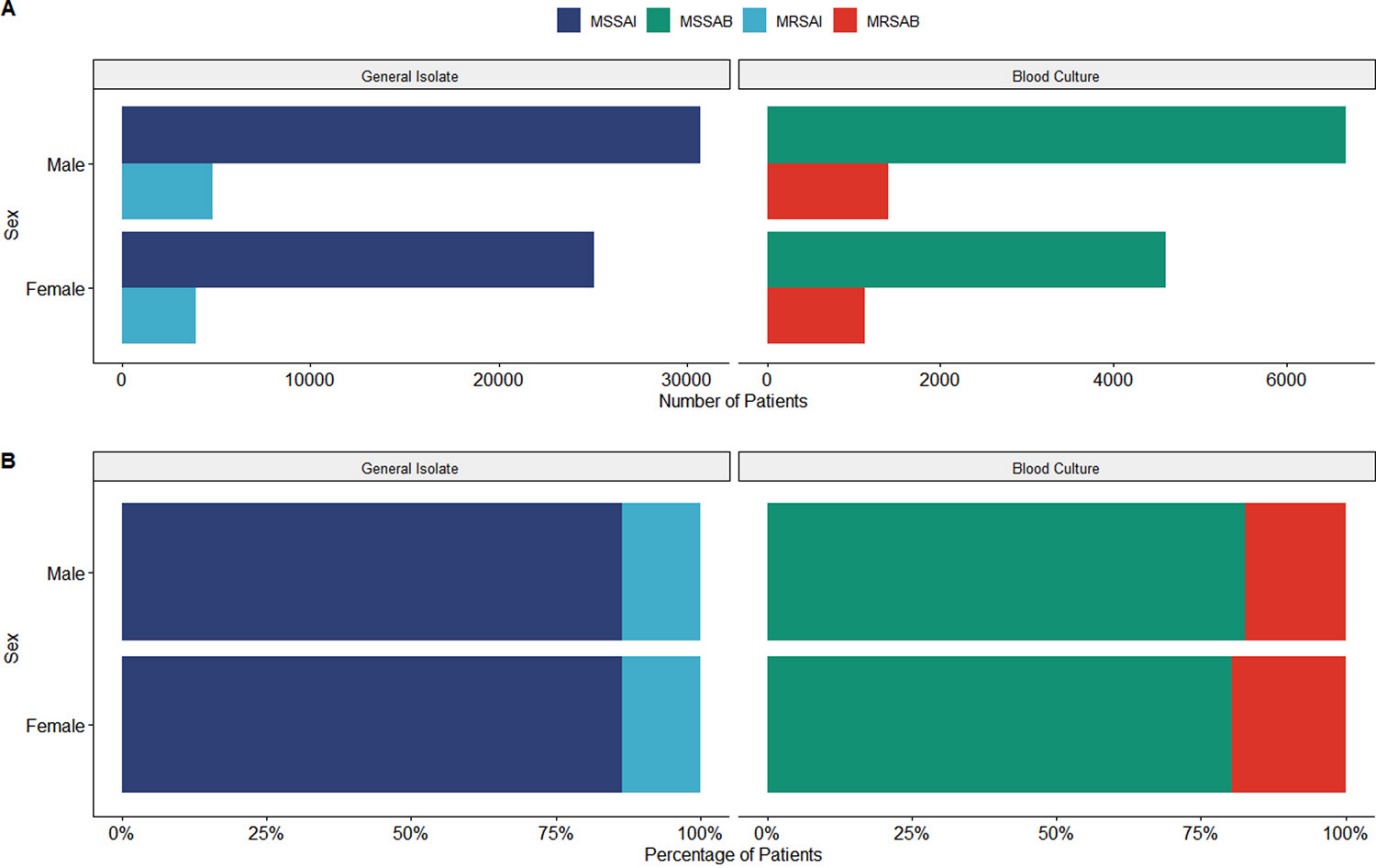
(A) Total number of cases by isolate type and patient sex split by site of origin. (B) Proportion of isolate types according to patient sex and split by the site of origin.

A male preponderance was seen, with 55.07% (95% CI, 54.68 to 55.45%) of general isolates and 58.50% (95% CI, 57.67 to 59.38%) of blood cultures. This preponderance did not have a significant relationship with resistance, as seen by a negligible interaction strength, V, of 0.00 (95% CI, 0.00 to 0.01; *P* < 0.001) for general isolates and 0.03 (95% CI, 0.01 to 0.05; *P* < 0.001) for bacteremia (Fig. 1).

**FIG 2:**
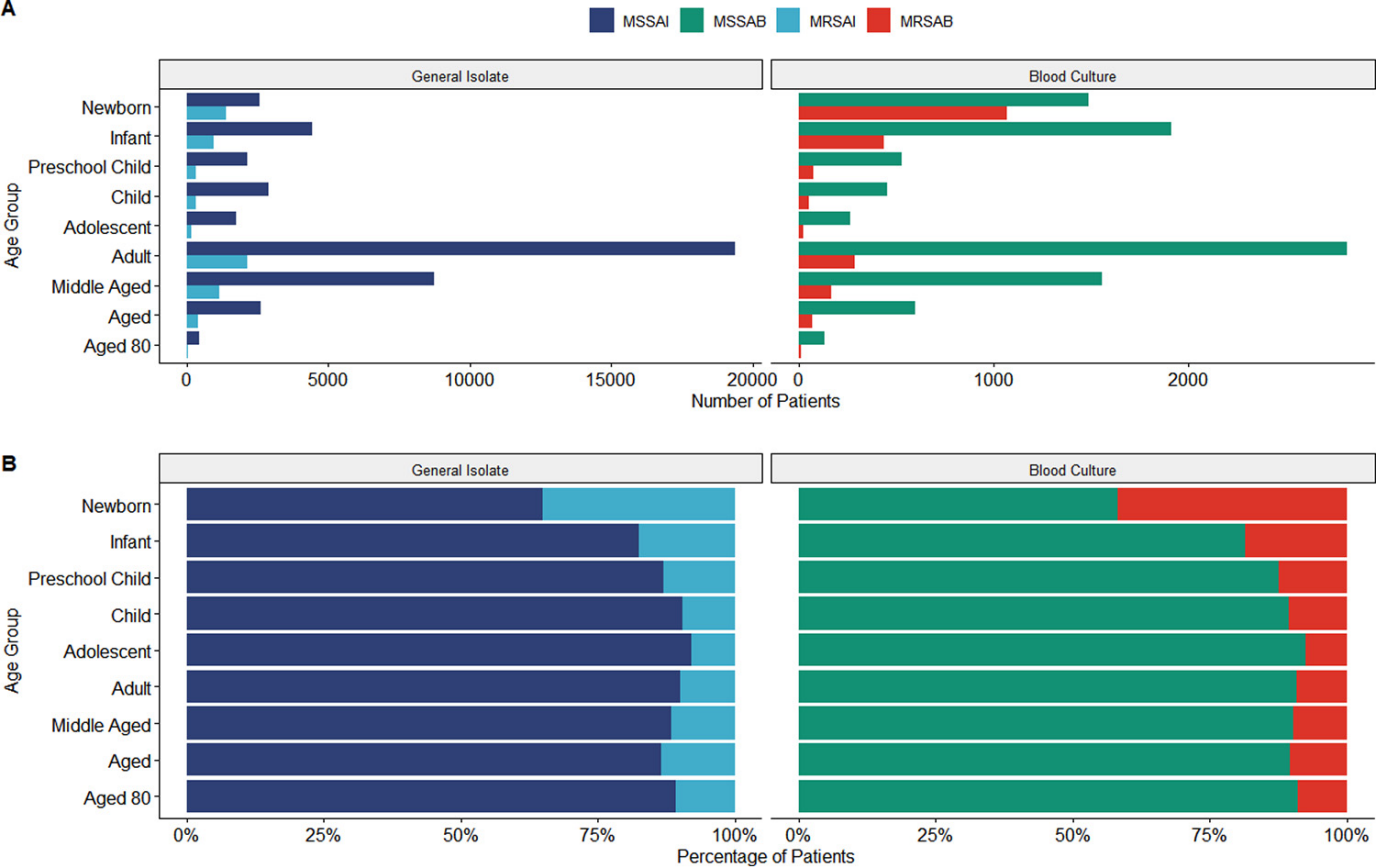
(A) Total number of cases by isolate type and patient age group split by site of origin. (B) Proportion of isolate types according to patient age group and split by site of origin.

General isolates had broadly similar age distributions between classes under a two-sided Kolmogorov-Smirnov test (D = 0.165; *P* = 0.073), whereas the distributions for bacteremia were significantly different (D = 0.289; *P* < 0.001). Adults bore the majority of sensitive bacteremia cases, 28.88% (*n* = 2,816), while most resistant bacteremia events occurred in patients under 5 years, 58.40% (*n* = 1,585) (Fig. 2). Among the patients under age five, the newborn group had the highest proportion of resistant bacteremia cases, 39.50% (*n* = 1,072). Age group categories had a moderate correlation with the type of infection for general isolates, V = 0.20 (95% CI, 0.19 to 0.21; *P* < 0.001). This correlation significantly increased for blood isolates, V = 0.33 (95% CI, 0.31 to 0.35; *P* < 0.001).

### Geographical locations.

**FIG 3:**
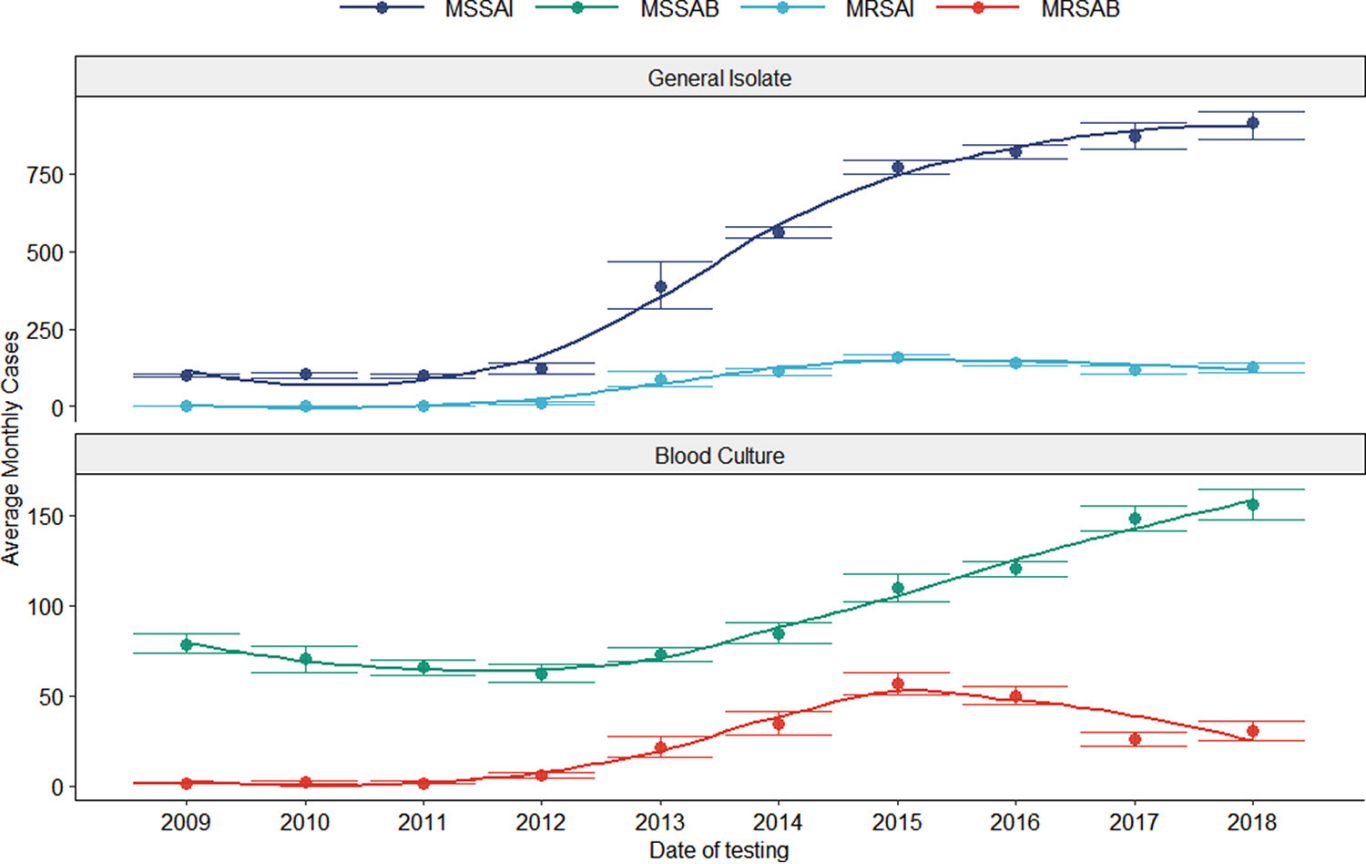
Average number of monthly infections per year in Gauteng from 2009 to 2018 with 95% confidence intervals subdivided by isolate type and split by the site of origin.

Geographically, among the 11 different provinces of South Africa, Gauteng is the most densely populated province and has been identified with the highest number of monthly infections per year from 2009 to 2018 in Gauteng (Fig. 3).

**FIG 4:**
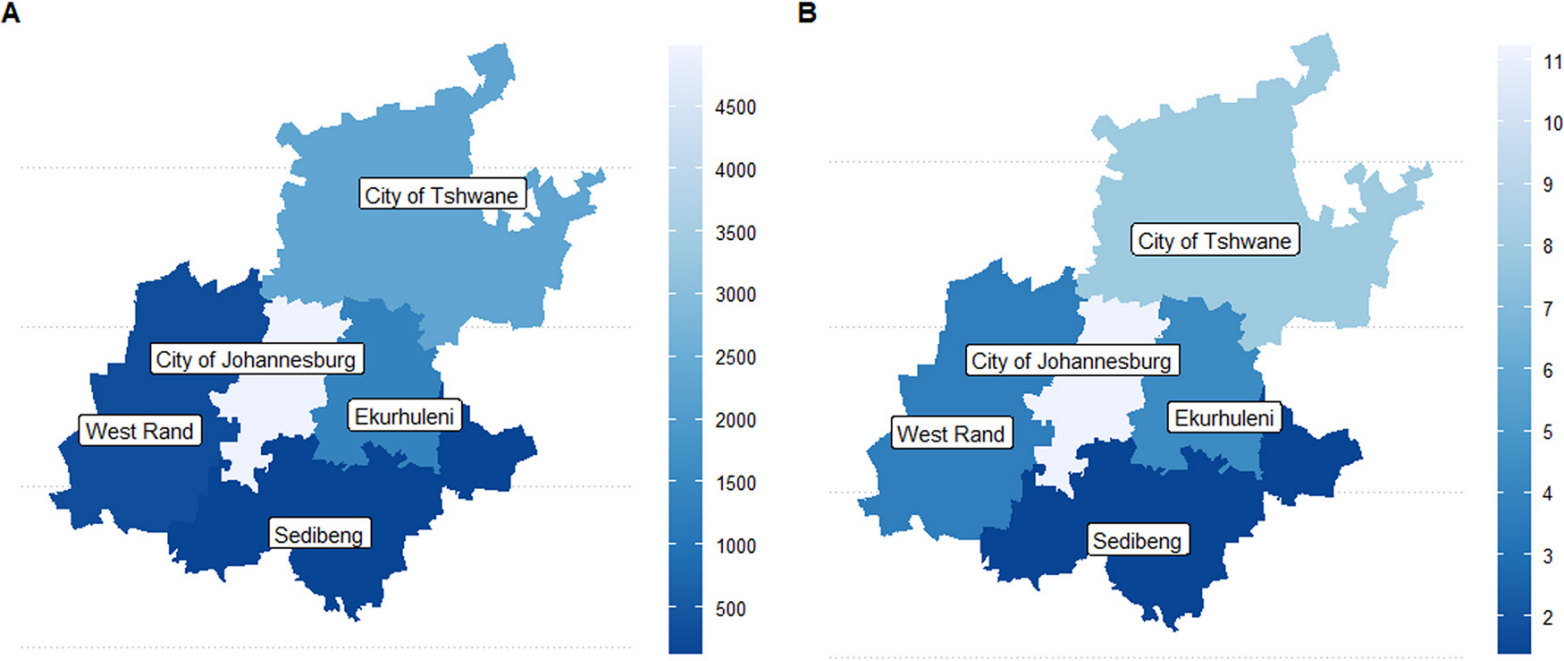
Total MRSA cases (A) and average infection rate per 100,000 (B) from 2009 to 2018 per Gauteng district.

Within the Gauteng Province, infections were substantially more common in the metropolitan municipalities (Johannesburg, Tshwane, and Ekurhuleni), with more than half the MRSA cases occurring in Johannesburg alone (MRSA isolates derived from any source [MRSAI], 54.5%; MRSA isolate subset from blood cultures [MRSAB], 55.2%) (Fig. 4). The cohort properties representing district and municipality locations of the S. aureus infections are presented in Table 2.

**TABLE 2:** Cohort properties of S. aureus infections based on district and municipality

Location	No. (%) of infections caused by^*a*^:	
	General isolates	Blood culture isolates
District		
City of Johannesburg	31,981 (48.4)	7,200 (50.2)
City of Tshwane	18,881 (28.6)	3,526 (24.6)
Ekurhuleni	8,027 (12.1)	2,140 (14.9)
Sedibeng	3,727 (5.6)	864 (6.0)
West Rand	3,455 (5.2)	626 (4.4)
Municipality		
City of Johannesburg	31,981 (48.4)	7,200 (50.2)
City of Tshwane	18,881 (28.6)	3,526 (24.6)
Ekurhuleni	8,027 (12.1)	2,140 (14.9)
Emfuleni	3,481 (5.3)	856 (6.0)
Lesedi	230 (0.3)	8*
Merafong City	697 (1.1)	9*
Midvaal	16*	0*
Mogale City	2,732 (4.1)	617 (4.3)
Rand West City	26*	0*

a *, percentages for these numbers were too low to report in this format.

Despite this clustering, district-level geographical information demonstrated an overall weak correlation with infection resistance for both general isolates, V = 0.10 (95% CI, 0.09 to 0.10; *P* < 0.001) and blood isolates, V = 0.12 (95% CI, 0.11 to 0.13; *P* < 0.001). This did not improve when using municipalities, as this subdivided the regions with the lowest case numbers and did little to alter the relationship.

### Ward distribution.

S. aureus bacteremia was substantially more common in medical wards, accounting for 56.69% (95% CI, 55.90 to 57.50%) of positive blood culture isolates, significantly more than all other wards surveyed. This distribution did not differ based on resistance, as seen by the proportion of isolates being similar (*t* = 0.79, *P* = 0.426). The cohort properties representing ward distribution of the S. aureus infections are presented in Table 3.

**TABLE 3:** Mean distributions of S. aureus infections in different wards

Ward type	Rate of MRSA (mean ± SD) among:	
	General isolates	Blood culture isolates
Medical	0.37 ± 0.48	0.57 ± 0.50
Surgical	0.41 ± 0.49	0.17 ± 0.38
OB-GYN	0.05 ± 0.23	0.04 ± 0.18
Outpatient	0.16 ± 0.36	0.06 ± 0.24
Pediatric	0.21 ± 0.41	0.38 ± 0.48
ICU	0.08 ± 0.27	0.17 ± 0.37
Casualty	0.07 ± 0.26	0.11 ± 0.31
Oncology	0.02 ± 0.13	0.02 ± 0.13

**FIG 5:**
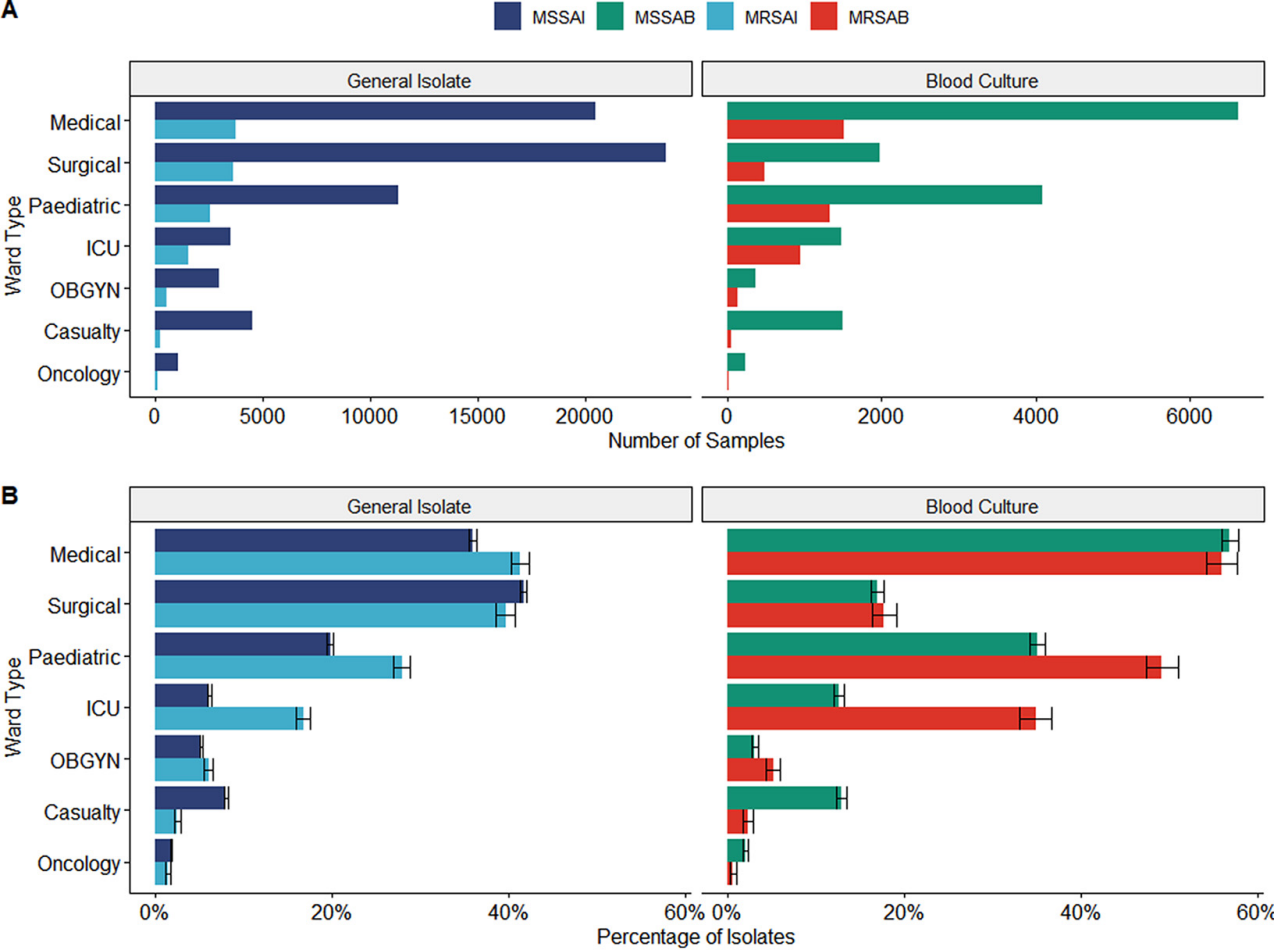
(A) Total number of cases by isolate type and ward type split by the site of origin. (B) Proportion of isolate types according to the ward type and split by site of origin.

In contrast to medical wards, surgical wards had a significant excess of general isolates compared with the number of bacteremia cases (*t* = −80.84, *P* < 0.001) (Fig. 5). Pediatric wards demonstrated the greatest absolute increase in resistant isolates, having only 28.05% (95% CI, 27.18 to 28.99%) of general resistant isolates while simultaneously having 49.12% (95% CI, 47.24 to 51.03%) of resistant blood culture isolates (*t* = 19.7; *P* < 0.001). Intensive care unit (ICU) wards had the most significant discrepancy in blood culture resistance, with a sensitive proportion of only 12.68% (95% CI, 12.08 to 13.25%), compared with an almost 3-fold-greater resistant isolate proportion of 34.97% (95% CI, 33.24 to 36.66%; *t* = 23.1; *P* < 0.001).

Casualty wards revealed the opposite bacteremia pattern, with resistant proportions of only 2.36% (95% CI, 1.84 to 2.91%) and a 5.5-times-greater sensitive proportion of 12.89% (95% CI, 12.31 to 13.49%; *t* = 24.7; *P* < 0.001). The excess of resistant cases for ICUs and excess of sensitive cases in casualty units were also demonstrated for general isolates to a lesser degree. Weak correlations were seen with bacteremia resistance for pediatric wards, V = 0.11 (95% CI, 0.10 to 0.13; *P* < 0.001), and casualty units, V = 0.13 (95% CI, 0.12 to 0.14; *P* < 0.001), whereas a moderate relationship was seen for ICU wards, V = 0.23 (95% CI, 0.21 to 0.25; *P* < 0.001). ICUs were the only units to demonstrate a nonnegligible correlation for general isolates, as they showed a weak relationship for infection class, V = 0.14 (95% CI, 0.13 to 0.15; *P* < 0.001).

### Infection rates.

Both MRSA and MSSA infection rates have been observed to change with time. The annual infection rates per 100,000 people are represented in Table 4.

**TABLE 4:** Yearly infection rate per 100,000 people, subclassed by isolate class

Yr	General isolates		Blood culture isolates	
	MRSAI	MSSAI	MRSAB	MSSAB
2009	0.08 (0.04–0.17)	10.42 (9.85–11.03)	0.07 (0.03–0.14)	8.16 (7.63–8.67)
2010	0.11 (0.06–0.21)	10.35 (9.78–10.95)	0.09 (0.05–0.17)	7.11 (6.65–7.61)
2011	0.11 (0.06–0.19)	9.66 (9.12–10.23)	0.09 (0.05–0.17)	6.43 (5.99–6.90)
2012	1.09 (0.91–1.28)	11.50 (10.92–12.12)	0.41 (0.31–0.54)	5.93 (5.52–6.37)
2013	8.18 (7.69–8.69)	35.88 (34.86–36.93)	1.99 (1.76–2.25)	6.74 (6.31–7.20)
2014	10.06 (9.53–10.61)	50.38 (49.19–51.60)	3.10 (2.81–3.41)	7.62 (7.16–8.10)
2015	14.05 (13.43–14.69)	67.59 (66.2268.99)	4.98 (4.61–5.36)	9.64 (9.13–10.17)
2016	11.95 (11.38–12.54)	69.99 (68.62–71.38)	4.25 (3.92–4.61)	10.28 (9.76–10.83)
2017	9.86 (9.36–10.39)	72.39 (71.02–73.80)	2.16 (1.93–2.42)	12.34 (11.77–12.93)
2018	10.18 (9.67–10.70)	74.03 (72.66–75.44)	2.50 (2.25–2.76)	12.68 (12.11–13.26)

Sensitive general isolate rates did not change significantly from 2009 to 2012; however, starting in 2013, significant year-on-year growth was seen until 2016. Resistant general isolates increased from 2013 only to minorly fluctuate for the remainder of the observed period. Sensitive bacteremia cases demonstrated an initial gradual decline from 2009 to 2012, which reversed from 2013 onwards with a progressive increase in case rates continuing for the remaining observations. Resistant bacteremia cases fluctuated from 2009 to 2012, followed by a growth in case rates from 2013. MRSA cases peaked between 2015 and 2016 and declined to a potential plateau from 2017 to 2018.

Subdivision of case rates by sex (Fig. 1A) replicated the overall change in cases while retaining the male preponderance over the entire period. Age-specific rates (Fig. 2A) revealed newborns accounted for 42.96% (95% CI, 41.31 to 43.68%) of the spike in 2015 MRSA bacteremia cases, with the remaining pediatric population contributing to 23.61% (95% CI, 21.48 to 25.57%) of cases. Resistant general and bacteremia cases have remained stable for adults and middle-aged individuals, with substantial growth in MSSA numbers since 2015.

District-specific analysis (Fig. 3) revealed the City of Johannesburg was responsible for 61.00% (95% CI, 59.51 to 62.18%) of the 2015 peak, with Ekurhuleni contributing the second-highest proportion of 21.55% (95% CI, 19.34 to 22.90%). The City of Tshwane was the only metropolitan not to have experienced a MRSAB spike in 2015. Ward-specific rate data (Fig. 4A) could not be utilized to ascribe case proportions, due to the one-to-many allocation of samples which results in sample duplication. Despite this, the wards demonstrating the most extraordinary resistant bacteremia case growth were the pediatric, ICU, and obstetrics and gynecology (OB-GYN) wards, which exhibited MRSAB peaks in 2015. The ICU MRSAB peak exceeded the rate of MSSAB acquisition, while pediatric and OB-GYN wards reached equivalent rates of resistant and sensitive bacteremia.

### Factor analysis.

**FIG 6:**
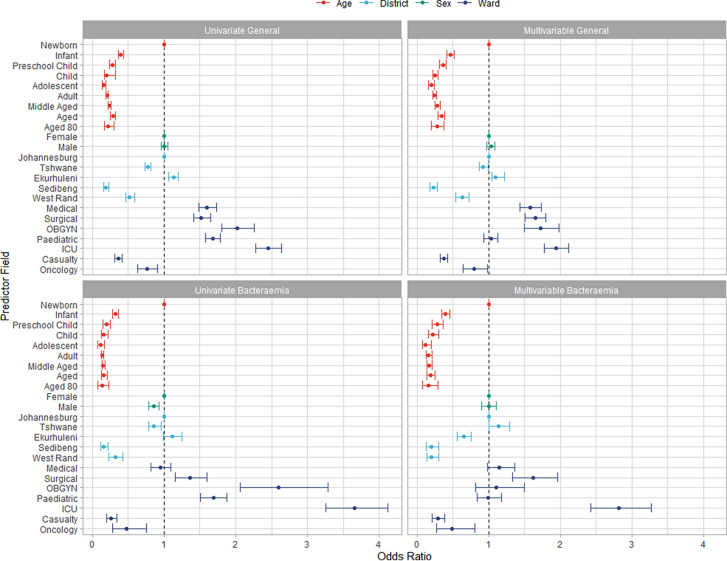
Factor analysis of monthly cases based on age, district, sex, and ward with 95% confidence intervals.

Logistic modeling of isolate resistance revealed significant discrepancies between associated features of general versus blood isolates. The overall factor analysis, including age, sex, district, and ward, is presented in Fig. 6. Age groups were significant associates across all models, irrespective of confounders. The elder groups tended to have lower odds ratios (ORs) for bacteremia compared to general isolates, with newborns as a baseline. This reflected the greater likelihood of acquiring resistant S. aureus bacteremia infections in earlier years of life. Sex associations demonstrated nonsignificant ORs except in the case of univariate bacteremia, but this resolved when confounders were considered for the multivariable case. District distribution interestingly showed a small but significantly increased propensity for resistant general isolates in Ekurhuleni compared to Johannesburg as the baseline. This occurred despite the district having a resistant bacteremia OR lower than that for Johannesburg. Tshwane, in comparison, exhibited an OR at least equivalent to the Johannesburg OR for resistant bacteremia but demonstrated a small but significant reduction in resistant general isolates.

Ward type estimates were quite different between sample types. Considering confounders, Medical, Surgical, OB-GYN, and ICU wards, there were significantly positive associations of general isolate resistance, while Casualty and Oncology units were significantly negative associations. Pediatric units were significantly associated with general isolate resistance in the univariate case but dissipated in the multivariable variant.

For bacteremia, surgical and ICU wards were the only significant positive associations of resistance, while Casualty and Oncology units showed significant negative associations in univariate and multivariable configurations. OB-GYN and Pediatric wards showed significant positive univariate associations; however, they were not significant when considering confounding factors. For OB-GYN units, this indicated that the propensity for resistance was likely explained by samples drawn from newborns within these units. ICUs appeared to have the greatest OR associated with resistance; however, age groups were the associations with the greatest strength. This can be seen by the newborn group OR of 6.25 (95% CI, 4.76 to 7.69) times the adult estimate, only appearing less important as the newborns were the baseline for age group comparisons.

## DISCUSSION

Staphylococcus aureus is one of the successful human pathogens that can cause a wide range of infections owing to its extensive virulence factors (1). Although S. aureus is one of the well-studied pathogens, there are visible knowledge gaps in understanding this pathogen's epidemiology. Given this, we conducted one of the most extensive studies to investigate S. aureus infections in South Africa. This study is the first to examine infections from all levels of health care, including clinics and secondary and tertiary health care centers.

All the collected and deduplicated S. aureus samples were considered general samples irrespective of acquisition source (blood, urine, swabs, etc.); about 22% were samples derived from blood cultures. This split into an overarching group and a bacteremia subgroup enabled broader analysis than is typically achieved through exclusive investigation of clinically significant cases. It can highlight all known resistant cases and the associated distribution across Gauteng. This further enabled an understanding of changing resistance patterns that may have preempted changes seen with only blood isolates. This may also have provided greater insight into the patient population susceptible to invasive resistant infections compared to the larger group, which may harbor MRSA without systemic involvement.

The minority of general isolates (13.79%) from this cohort were MRSA, which is concordant with the findings of Oosthuysen et al. (14), where only 15.3% of isolates obtained from any source at Tygerberg Hospital were found to be MRSA. However, many other studies in South Africa and globally have found much higher percentages of MRSA in comparison to MSSA when studying S. aureus isolates (8, 15, 16). Previous South African studies found the proportion of resistant isolates to be as high as 30 to 46%, which differs greatly from this study’s finding of 13.79% and 18.91% for general and blood isolates, respectively (8, 9, 13). Diekema et al. (7) found that MRSA isolates accounted for 40% of S. aureus samples in an international study carried out across 45 countries. Fluctuations of both MRSA and MSSA have been reported by several studies, and it is evident that infection rates of both MSSA and MRSA generally mirror one another (17). This disparity is likely an aliasing phenomenon due to studies occurring at different phases of MRSA case growth, and this study examining a rather broad period. The MRSA proportion, for example, goes up to 34.05% of blood samples when considering only the peak in 2015, which is closer to the estimates of the studies around this period. This was not replicated for general isolates, as the proportion only increased to 17.21% for the same 2015 period. Other potential causes of this disparity could be because most of the previous studies were carried out only in hospitals (and more specifically, tertiary and academic hospitals). In contrast, the data set considered in this study included all samples from Gauteng Province processed by the NHLS. Rural district hospitals in South Africa may be more likely to empirically treat patients without waiting for blood cultures to be performed, due to poor access and long waiting times. This may explain why this study’s figures and those of previous studies differ in terms of MRSA preponderance.

Falagas et al. described a decrease in MRSA prevalence in South Africa between 2006 and 2011 (6). This is contrary to the findings of this study, where a general upward trend of MRSAI was found from 2009 onwards. Infection rates peaked between 2015 and 2016, with a downward trend after that. Perovic et al. demonstrated a much earlier drop in MRSA cases between the years 2010 (53%) to 2012 (40%) (8). This drop in MRSA cases was attributed to better infection control and antimicrobial management. Similarly, the decrease in infection rates in Gauteng after 2015 may be attributed to the fact that South Africa developed the Antimicrobial Resistance National Strategy Framework in 2014, an effort to decrease antimicrobial resistance in the country. Though there was a period of substantial growth in the number of MRSA and MSSA cases between 2013 and 2016, it is evident that MRSA and MSSA have stabilized and even decreased, as seen in the rates of MRSAB after 2017. Whether these rates have plateaued in recent years or have declined is unclear. Furthermore, the international peak of infection rates with MRSA was between 2005 and 2008 (7), much closer to the local peak described by Perovic et al. (8). Our study, which covered many more infection episodes than other local studies, found that the rise of MRSA infection was, in fact, later than the international peak, between 2015 and 2016, as mentioned above.

When taking a closer look at the demographic data obtained, a male predominance was found in MRSA and MSSA. This is similar to findings at Tygerberg Hospital (in Cape Town, South Africa), where 59.4% of those studied with S. aureus were male (14). Perovic et al. and Fortuin de Schmidt et al. have also shown male predominance in previous studies (8, 13). Although males were more likely to get both MRSA and MSSA found in general and blood isolates, the preponderance of MRSA in infected individuals was not different between sexes.

This study also reports that the highest percentage of MRSA bacteremia (39.5%) was among newborns, whereas newborns accounted for a much smaller proportion of MSSA bacteremia. These results are congruent with previous findings where children under 9 years old accounted for most S. aureus infections (2, 8, 13). Also, it has been observed that adults account for the highest number of general isolates in both MRSAI and MSSAI, which is supported by the previous findings of Shuping et al., who reported adults accounted for the highest percentage of MSSA infections (9). Notably, older adults did not demonstrate an increased proportion of infection. This contradicts previous findings, which showed that those at extremes of age had higher infection rates (8).

Johannesburg had more cases of both general and blood isolates of MRSA than other geographical locations, similar to previous studies (13). MSSA infections in this area were also higher than in other regions, although previous studies did not investigate this. Many infective episodes in Johannesburg could be because this is a highly population-dense area.

Patients who have not recently undergone surgery and are diagnosed with bacteremia are much more likely to be treated in a medical ward. This accounts for why patients treated in medical wards accounted for the highest preponderance of bacteremia. Fortuin de Schmidt et al. found that 60% of the patients presenting for S. aureus infections had one or more predisposing conditions (13). This may serve as another explanation for why bacteremia events were higher in medical wards. Patients may have been admitted for a different medical condition which could have predisposed them to S. aureus bacteremia. The subtype found most in surgical wards was general isolates of MSSA. This may be due to skin commensals infecting surgical sites. Skin colonization with S. aureus is most commonly associated with MSSA instead of MRSA, which is in keeping with this study’s findings. Interestingly, surgical patients accounted for very few of the bacteremia cases. The physiological stress of surgery and surgical procedures would seemingly predispose patients to bacteremia. Prophylactic antibiotics before or during surgery may have minimized the risk of bacteremia. Alternatively, after surgery, patients with S. aureus bacteremia may have also presented with a surgical site infection that could have been sampled and cultured without blood cultures being performed. This would have been logged as a general isolate, not a blood isolate. On the other hand, more cases of MRSA bacteremia were seen in ICU and Pediatric wards than other subtypes of S. aureus. This may have been due to the immune status of these patients being such that it is vulnerable to bacteremia.

### Conclusion.

Multiple conclusions can be drawn from this research. MRSAB rates were increasing and peaked in Gauteng in 2015. Since then, they have started to decrease and maybe plateau, but further years need to be analyzed to determine if this is the case. The high number of cases in children under 5 years of age emphasizes the need to improve infection prevention and control measures in pediatric populations and pediatric wards in Gauteng hospitals. MRSA infections are still dominating the MSSA cases, and therefore use of antibiotic stewardship principles needs to be strongly recommended. The demographic, geographic, and site information is valuable, as it informs the areas in which changes should be implemented.

Overall, this study emphasizes the value and importance of ongoing surveillance of MRSA infections in Gauteng. Surveillance of antimicrobial usage for MRSA and MSSA infections is also necessary. Recommendations for further research include analyzing the trends of antibiotic resistance in this Gauteng population over time and continuing to model the MRSA trends in Gauteng in the years following this study.

## MATERIALS AND METHODS

### Study design.

This study was carried out as a retrospective, descriptive analysis of the data of all S. aureus isolates obtained from patients at all public hospitals in Gauteng that were processed by the South African National Health Laboratory Service (NHLS) from 01 January 2009 to 31 December 2018.

### Data collection.

The data set collected contained all S. aureus isolates, either MSSA or MRSA, from any biological source received by the NHLS from January 2009 to December 2018 from any facility located within Gauteng, South Africa. Samples with missing or invalid patient details and samples without identification codes were excluded, as were any samples received that were collected outside this period. The data set included related patient data such as demographics (age, sex, and ethnicity), geographical origin (ward, hospital, and district), and the type of sample used (blood, swab, sputum, etc.). The data were initially deduplicated by grouping samples submitted together as a single set. Samples containing both MRSA and MSSA isolates were treated as separate events. Isolates derived from any source were labeled as all isolates (MRSAI or MSSAI), while a subset of isolates derived from blood cultures was labeled as bacteremia (MRSAB or MSSAB). Isolates were defined as belonging to the same infectious episode if detected within 30 days of a previous positive result, with results beyond this being flagged as a recurrent infection. All isolates detected as belonging to the same episode were collapsed to a single record.

### Sources of bias.

The data set was drawn from laboratory samples performed on clinician request. This may have resulted in underestimating the incidence due to unrecognized cases or those treated empirically without laboratory confirmation. Demographic and geographical data were largely transcribed from written records, which may have been incorrect or incomplete and could not be externally verified.

### Age groups.

Patients were divided into groups based on their ages following Medical Subject Headings (MeSH) definitions (18). Age groups were grouped for trend analysis into neonate, pediatric, adult, and elderly groups, as described in Table 5. The similarity between the distributions of patient ages across isolate classes was assessed using the Kolmogorov-Smirnov test.

**TABLE 5:** Age group definitions following MeSH definitions

Age group	MeSH definition	Trend group
Newborn	Birth to 1 mo	Neonate
Infant	1–23 mo	Pediatric
Preschool child	2–5 yrs	
Child	6–12 yrs	
Adolescent	13–18 yrs	
Adult	19–44 yrs	Adult
Middle-aged	45–64 yrs	
Aged	65–79 yrs	Elderly
Aged 80 and over	80 yrs and over	

### Geographical distribution.

Five districts and nine municipalities within Gauteng Province were identified and utilized to visualize the geographical distribution of cases via choropleth maps. The wards where isolates were collected were contacted to ascertain the associated medical discipline(s) and classified in a one-to-many fashion. Each ward could belong to multiple disciplines simultaneously, i.e., a pediatric medical ward would be allocated to both medicine and pediatrics. The wards were classified as Medical, Surgical, Pediatric, OB-GYN, ICU, Casualty, and Oncology.

### Annual comparisons.

Yearly average case figures were derived by determining each year's overall monthly average case rate and the associated 95% confidence intervals through bootstrapping the mean. Yearly comparisons were made using monthly isolate granularity. Incident figures were determined at the district and municipal levels via provincial population estimates derived from the 2011 South African census (19), with yearly changes in population estimated by national population growth figures.

Logistic regression was utilized to determine ORs for variables associated with drug-resistant infections. All variables were prespecified as informed by previous literature findings and included within both univariate and multivariable analyses. A cross-validated Lasso regression model was additionally employed for confirming the utility of the selected variables (20). Categorical variables were one-hot coded for model construction except for ward type. Owing to the multilabel nature of this field (e.g., a medical pediatric ward), the variable was full rank encoded to enable any combination of ward types to be addressed.

### Statistical analysis.

A chi-square and Cramer's V tests assessed evidence of the correlation between sample class and categorical variables. These provided a measure of significance and quantified the effect size associated with the correlation (21). Statistical significance was considered with an α of <0.05. The quantification of effect size was necessary given the size of the data set, as standard chi-square analysis is substantially more likely to produce seemingly significant results with larger sample sizes irrespective of the proper relationship between variables (22). Cramer’s V values were interpreted as shown in Table 6 (23). Comparisons between proportions were made using the Student's *t* test or Wilcoxon test, depending on the expected normality of the data to be compared.

**TABLE 6:** Interpretation of Cramer's V values

Cramer's V value range	Interpretation of correlation
0.00–0.10	Negligible
0.10–0.20	Weak
0.20–0.40	Moderate
0.40–0.60	Relatively Strong
0.60–0.80	Strong
0.80–1.00	Very Strong

### Ethical considerations.

This study was approved by the Human Research Ethics Committee (Medical) at the University of the Witwatersrand, Johannesburg (protocol number M190837). This study received a waiver of informed consent, as the analysis is retrospective and of minimal risk to participants. The data were collected during routine patient care; therefore, this study was unlikely to affect participants adversely. In addition, the data received were anonymized and securely hosted via an authentication-based cloud hosting solution only accessible to research collaborators. The analysis of further anonymized samples provided only aggregate statistical information, with geographical data granularity limited to the subdistrict level.
